# MDR Pumps as Crossroads of Resistance: Antibiotics and Bacteriophages

**DOI:** 10.3390/antibiotics11060734

**Published:** 2022-05-30

**Authors:** Pavel A. Nazarov

**Affiliations:** Belozersky Institute of Physico-Chemical Biology, Lomonosov Moscow State University, 119991 Moscow, Russia; nazarovpa@gmail.com

**Keywords:** bacteriophage, antibiotic, MDR pump, AMR resistance, antibiotic alternative, gram-negative bacteria, gram-positive bacteria, efflux, phage receptor

## Abstract

At present, antibiotic resistance represents a global problem in modern medicine. In the near future, humanity may face a situation where medicine will be powerless against resistant bacteria and a post-antibiotic era will come. The development of new antibiotics is either very expensive or ineffective due to rapidly developing bacterial resistance. The need to develop alternative approaches to the treatment of bacterial infections, such as phage therapy, is beyond doubt. The cornerstone of bacterial defense against antibiotics are multidrug resistance (MDR) pumps, which are involved in antibiotic resistance, toxin export, biofilm, and persister cell formation. MDR pumps are the primary non-specific defense of bacteria against antibiotics, while drug target modification, drug inactivation, target switching, and target sequestration are the second, specific line of their defense. All bacteria have MDR pumps, and bacteriophages have evolved along with them and use the bacteria’s need for MDR pumps to bind and penetrate into bacterial cells. The study and understanding of the mechanisms of the pumps and their contribution to the overall resistance and to the sensitivity to bacteriophages will allow us to either seriously delay the onset of the post-antibiotic era or even prevent it altogether due to phage-antibiotic synergy.

## 1. Time of Global Crisis: Antibiotic Resistance

Antimicrobial resistance is now threatening the very foundations of modern medicine and has implications for all areas of health [1]. This has become especially important in connection with the SARS-CoV-2 coronavirus pandemic, when diseases of different and mixed etiologies can serve as a trigger to accelerate the formation of antibiotic-resistant clinical strains. The globalization of the world economy, in addition to its positive aspects, also has negative effects, such as an increase in the rate of infection transmission from one point of the globe to another. We observe this in the example of the spread of new strains of coronavirus, such as omicron [2], but this is not limited to the world of viruses, as bacteria, including multi-resistant ones, spread just as quickly. For example, the gene blaNDM-1, which was discovered in India subcontinent in 2009 [3] reached a remote region of the Arctic, Western Svalbard [4], and the gene for resistance to the antibiotic colistin, mrc-1, which was discovered in China in 2015 [5], has been detected in 2018 at least in 31 countries on 6 continents, including in the U.S., Brazil, South Africa, Russia, Vietnam, Japan and Germany [6].

At the moment, the development of new antibiotics is practically suspended by big pharma for several reasons. Although there are several antibiotics in the development pipeline [7,8], clinical phase development remains narrow, consisting largely of derivatives from known antibiotics [9]. There are several major ways to search for new antibiotics: (1) looking for antibiotics among cultivated microorganisms; (2) enumeration of libraries of chemical compounds with subsequent modeling of the location (molecular docking) of a promising structure in the active center of the proposed target; (3) targeted synthesis of hybrid chemical compounds to deliver antibiotics or to disrupt the bioenergy/integrity of bacterial membranes; and (4) looking for antibiotics among non-culturable microorganisms [10]. Unfortunately, each of the ways to find new antibiotics has weaknesses that make such research expensive and risky.

Thus, the search for antibiotics among cultivated organisms is complicated by the fact that approximately one million of new actinomycetes need to be analyzed in order to find only one new antibiotic [11], and this greatly increases the cost of research and makes it comparable to searching through libraries of chemical compounds. However, spontaneous mutations in bacteria, especially as a result of improperly administered antibiotic therapy or the misuse of antibiotics in agriculture, can wipe out several years of laboratory work in a short time. Although there are successful examples of the creation of effective hybrid antibiotics to disrupt the bioenergetics [12,13,14,15] of membranes or to deliver other antibacterial substances [16,17], this approach may have its limitations (for example, toxicity to eukaryotic cells, often explained by the detergent action of the substances used [18]) although this has not been directly shown to occur in the range of effective concentrations. The search for antibiotics among previously uncultivated microorganisms yields successful results [19,20,21], but this may not be enough for the needs of modern medicine.

The fact that the modern direction of the search for new antibiotics is experiencing a serious crisis is not a secret for anyone. In the past 15 years, only one new class of antibiotics against gram-positive bacteria has been introduced into clinical practice. The last class of broad-spectrum antibiotics was introduced into the clinic in the 1960s [22]. Thus, humanity is on the verge of a global crisis and can be thrown back into the pre-antibiotic era.

Therefore, along with the search for new antibiotics, it is necessary to find alternative approaches to the treatment of bacterial infections. Despite the fact that there is a fairly large variety of approaches to obtaining effective alternatives to antibiotics and they have been applied for more than a decade, there have been no bright breakthroughs in the treatment of infections with their help. Only a few effective alternatives to antibiotics exist, indicating the still higher efficacy of antibiotic therapy.

The most interesting alternatives are as follows: vaccines [23], antibodies [24], probiotics [25], immunostimulants [26], photosensitizers [27,28], bacteriophages [29], phage lysins [10,30,31], engineered bacteriophages [32], antimicrobial peptides [33], host-protecting peptides [34], antibiofilm compounds [35], multidrug resistance pump inhibitors [36,37], immunosuppressants (combinatorial action with antibiotics in transplantology) [38], liposomal toxin traps [39], metal chelators [40,41,42], antibacterial nucleic acids [43,44,45]. Without a doubt, bacteriophages and their products (lytic enzymes, phage holins) are the most promising and deserve the closest attention [46,47,48].

## 2. Antibiotic Resistance: The Role of MDR Pumps

Antimicrobial resistance is a major global problem that could lead to 10 million annual deaths by 2050 [49]. Therefore, much attention is now paid to understanding the mechanisms of antibiotic resistance and overcoming them. The mechanisms of protection of bacteria from antibiotics are complex and diverse, and can be caused by both genetic factors and the physiological state of the bacteria themselves. These mechanisms may be natural for a particular microorganism or acquired from other microorganisms.

**Figure 1:**
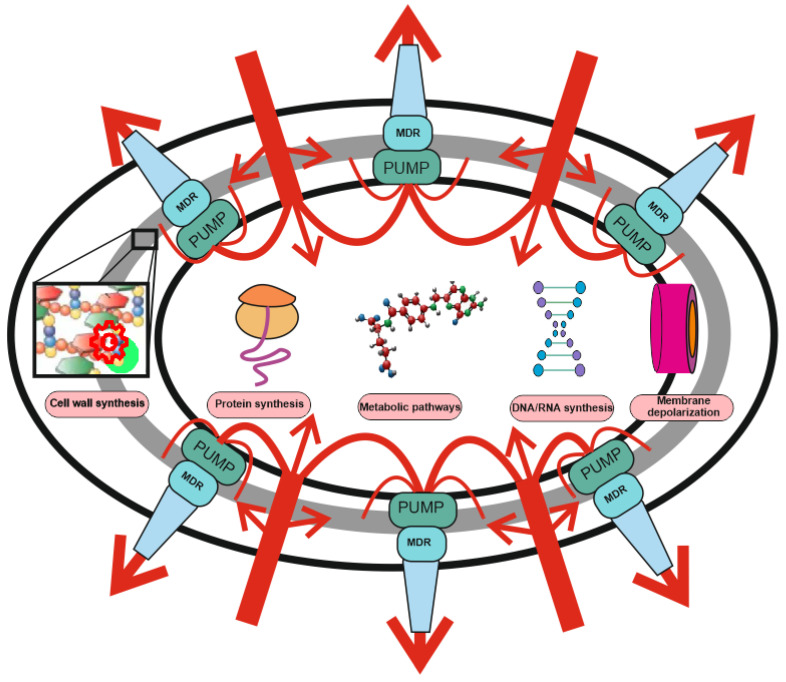
Two-level protection of bacteria from antibiotics: the role of MDR pumps in antibiotic resistance. Protection against antibiotics includes non-specific protection due to the operation of combination of MDR pumps with a wide range of substrate specificity (level 1), and specific protection (level 2), including drug or target modification, drug inactivation, target switching and target sequestration. Antibiotic uptake (red arrows) is significantly weakened or stopped under the action of non-specific protection by means MDR pumps’ composition and only a small part of the drugs reaches the targets (protein synthesis, DNA/RNA synthesis, membrane depolarization, metabolic pathways, and cell wall synthesis). The efflux pump contribution may not be visible, but it can increase the MIC by several orders of magnitude. MDR pumps are involved in resistance to antibiotics with any mechanism of action and protect bacteria even against those antibiotics for which they do not have specific resistance mechanisms.

The main mechanisms of resistance are considered to be: (1) drug uptake restriction, (2) drug target modification, (3) drug inactivation and (4) active drug release, (5) target switching, and (6) target sequestration [22,50]. At the same time, the role of some resistance mechanisms is often overestimated at the expense of others (See Figure 1).

An illustration of this is the transformation of SkQ1 (10-(6′-Plastoquinonyl)decyltriphenylphosphonium) from the mitochondria-targeted antioxidant and “non-antibiotic” [51] into the most effective antibiotics with one of a deep-studied mechanism of action. SkQ1 is an ideal tool for studying MDR pumps, since it acts on bacterial bioenergetics and, being in the membrane, reduces the membrane potential of bacteria. The only protection against such a substance is the operation of the MDR pumps.

It was believed that the sensitivity of bacteria to SkQ1 is determined by the complexity of the cell wall, and Gram-positive bacteria with a more simply arranged cell wall are sensitive to it, while Gram-negative bacteria with a complex cell wall are resistant. This was explained by the difficulty of SkQ1 penetration through the double membrane of gram-negative bacteria, thus, resistance was reduced only to the ability to hinder the penetration of the substance into the bacterial cell [13]. However, further studies showed [14] that deletion of any of the AcrAB-TolC pump proteins (AcrA, AcrB, or TolC), with the exception of the small protein AcrZ [15], led to a complete loss of resistance to SkQ1 in deletion mutants, which completely changed the concept of resistance. Obviously, in this case, the resistance turned out to be independent of the complexity of the cell wall and the very ability of the substance to penetrate through cell membranes, as previously thought, and was dependent solely on the presence of the AcrAB-TolC pump. It seemed that the resistance of *Escherichia coli* bacteria and the sensitivity of other gram-negative bacteria are associated precisely with the presence of this pump however, everything turned out to be even more complicated. All SkQ1-sensitive gram-negative bacteria have an AcrAB-TolC pump in their cell envelope, which seemed to contradict the hypothesis. Only the analysis of the AcrB protein sequences of the AcrAB-TolC pump made it possible to explain everything. The protein sequences of resistant bacteria, for example, *Klebsiella pneumoniae*, were close to that of *E. coli*, while they differed by 35–60% in sensitive bacteria, which made it possible to formulate a functional criterion for evaluating MDR homologous pumps [52]. Obviously, in the process of research, there was a reassessment of the mechanisms of resistance to SkQ1 from the mechanism of “limitation of drug absorption” to the mechanism of “active release of the drug”, which indicates a previous underestimation of the mechanism of antibiotic efflux which apparently required a reevaluation.

The significant contribution of the “drug absorption limitation” mechanism is apparently determined in large part by insufficient investigation of a large number of antibiotics and the pleiotropy of the action of pumps, when many pumps can pump out one antibiotic and the removal of even a few pumps has little effect on the overall effect of antibiotic outflow and conventional microbiological methods are not capable of detecting them adequately. Looking at the antibiotic efflux sensitivity profile of *E. coli* [53], one can see that the “active drug release” mechanism leads to a 50–100-fold increase in the minimum inhibitory concentration for antibiotics, and we observe the same order of magnitude in the case of SkQ1 [14]. Thus, MDR pumps appear to play a key but underestimated role in antibiotic resistance.

To understand how great this influence is, it is necessary to pay attention to the main mechanisms of action of antibiotics. Table 1 presents the main types of antibiotics and the effect of MDR pumps on bacterial resistance to them. Not surprisingly, antibiotics that affect the biosynthesis of proteins, DNA, RNA or disrupt metabolic processes, such as folate synthesis, for which penetration is a necessary condition for antibacterial action, demonstrate a dependence of resistance on pump operation [53,54,55,56,57,58,59,60,61,62,63,64]. However, pumps having any effect on antibiotics that do not penetrate into the cell cytoplasm, but are localized on the membrane, or even beyond it [65,66,67,68,69], would cause sincere surprise if it were not for the fact that some pumps expel their substrates from the periplasmatic space [70]. Thus, MDR pumps are a universal tool that protects the bacterial cell itself and its microenvironment from the negative effects of xenobiotics.

## 3. Alternative Resistance Mechanisms Using MDR Pumps

Bacteria use their MDR pump system to effectively defend against various antibiotics and toxins. Moreover, they use various approaches to improve its effectiveness. Resistant phenotypes can arise as a result of increased pump activity due to their overexpression, as is observed with the addition of sublethal concentrations of antibiotics, which, through a cascade of feedback-driven interactions, causes an increase in the expression of MDR pump genes that evacuate these antibiotics [71]. We observe a similar approach in the case of unicellular eukaryotes [72], which indicates a universal defense mechanism.

In addition, pumps may contribute to different ways of expelling antibiotics, but cooperate to work together, efflux antibiotics and provide an even higher level of protection than necessary [73,74,75]. This fact indicates that a large concentration of antibiotics is required to overcome the resistance from MDR pumps, and even without special protection mechanisms, bacteria are well protected from negative effects only due to the action of MDR pumps.

Another interesting aspect is the asymmetric accumulation of MDR pumps during cell division, when the pumps are mainly located at the old poles, and new poles are newly created and MDR pumps are synthesized de novo [76]. This creates a variable resistance profile during the cell cycle, which makes it possible for a population to contain bacteria with different expression status of MDR pumps. When encountering an antibiotic, the least resistant ones die and are used in two mechanisms: (1) adsorption of antimicrobials on the surface of dead cells, thereby protecting the remaining bacteria [77,78]; and (2) secretion of a “necrosignal” that causes the activation of protective pathways in surviving bacteria [79]. As a result, the bacterial population acquires increased resistance to antibiotics.

Undoubtedly, an interesting mechanism is the increased rate of gene mutation in bacteria under the action of antibiotics [80,81], which can lead to a protective effect, including mutations in MDR pumps and heterogeneity of their expression [82]. Given that gene copy number change is a common event in all genomes [83], doubling the MDR pump genes results in an increased chance of survival when antibiotics are added. Thus, gene duplication leads to an increase in resistance, and may be an alternative to changing the level of pump expression.

Thus, we can confidently conclude that bacteria use to the fullest extent all of the opportunities presented to them by MDR pumps to increase resistance and survive in the presence of antibiotics in the environment or even in the case of antibiotic therapy.

## 4. MDR Pumps in Bacteria

**Figure 2:**
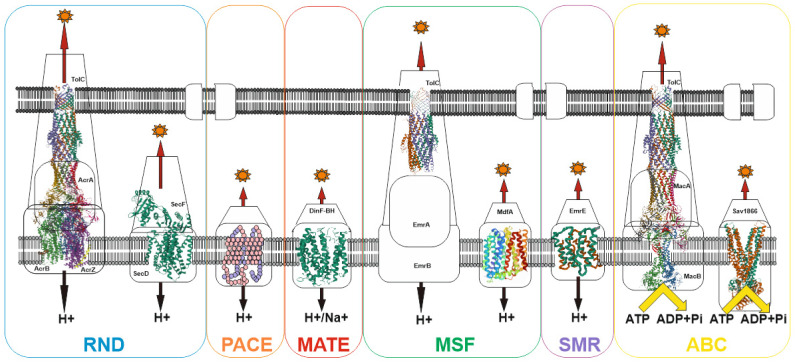
Schematic of representative major superfamilies of bacterial multidrug transporters and tripartite assemblies. Structures of representatives of each of the transporter families (except PACE) are presented. Protein Databank (PDB) identifiers from left to right: AcrABZ-TolC (5O66), SecDF (3AQP), DinF-BH (4LZ6), TolC (2XMN), MdfA (4ZOW), EmrE (3B62), MacAB-TolC (5NIL), and Sav1866 (2HYD). The structures of the proteobacterial antimicrobial compound efflux (PACE) class of transporters have not yet been experimentally resolved and are therefore represented here as a basic outline.

Despite the wide variety of xenobiotics, MDR bacterial pump systems belong to six large groups: (1) the ATP-binding cassette (ABC) family; (2) the proteobacterial antimicrobial compound efflux (PACE) family; (3) the major facilitator superfamily (MFS); (4) the multidrug and toxin extrusion (MATE) family; (5) the small multidrug resistance (SMR) family; (6) the resistance-nodulation-cell division (RND) family [84]. Most of the families have an early origin and have been preserved in the course of evolution, as evidenced, for example, by the ubiquitous distribution of the MFS and RND families both among prokaryotes and eukaryotes [85,86]. Currently, the recently discovered proteobacterial antimicrobial compound efflux (PACE) family remains the least studied family [87]. Among other well-studied transporter families, only the ATP-binding cassette (ABC) family uses energy in the form of ATP to do the job of substrate efflux. For all other families, the energy of the proton (or H+/Na+) gradient is used to perform work (See Figure 2).

This apparently indicates that two main states can exist for a bacterial cell: (1) absence of potential on the membrane but presence of ATP, when only the transporters of the ATP-binding cassette (ABC) family work; (2) the presence of a potential on the membrane and, accordingly, ATP, in which case all transporters work. Since the membrane potential is associated with ATP generation, the absence of ATP in the presence of the potential can be excluded. Thus, voltage-dependent pumps predominate in prokaryotes, which is explained by the high potential on the membrane (~140–220 mV for *E. coli* [88]) and the absence of the need to convert the potential into ATP. This provides an advantage in the pumping rate. In eukaryotes, the situation is slightly different: the main pumps are ATP-dependent, since ATP generation and a high membrane potential (~180 mV [89]) occur in mitochondria, and thus the energy of the membrane potential cannot be directly used to perform work. Hence, the combination of voltage-dependent and ATP-dependent pumps determine the resistance profile of bacteria.

**Figure 3:**
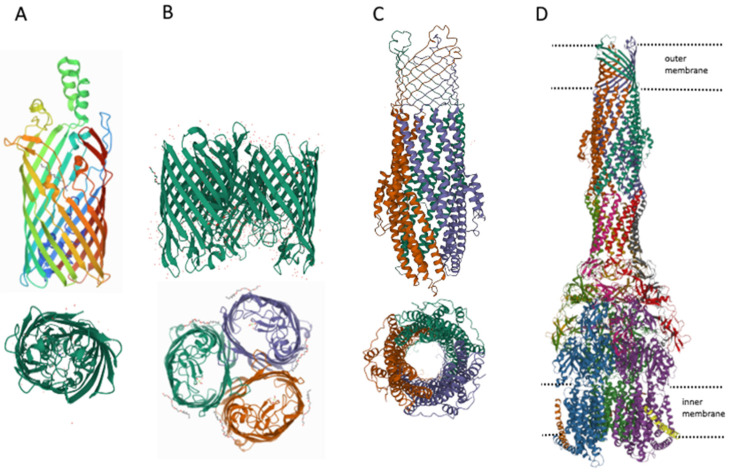
Structures of outer membrane proteins FadL (**A**), OmpF (**B**), TolC (**C**), and transporter AcrABZ-TolC (**D**). PDB identifiers for FadL (2R88), and OmpF (6ZHP).

Gram-negative bacteria differ from gram-positive bacteria by the presence of an outer membrane [90], which serves as an additional barrier preventing the penetration of substances into the bacterial cell. The main features of the permeability of gram-negative bacteria are well described in [91], therefore, in summary, we would like to note that there are three main ways of penetration of substances through the outer membrane: (1) penetration due to porins (see Figure 3), for example, OmpF, OmpC; (2) transfer by specific channels (e.g., FadL); and (3) transfer of lipophilic compounds by flip-flop across the lipid component of the membrane.

However, it can be imagined that there is at least one other transport channel, the outer membrane protein TolC. The TolC protein is a component of eight MDR pumps: the most well-studied main pump, AcrAB-TolC, and other pumps, AcrAD-TolC, AcrEF-TolC, MdtABC-TolC, MdtEF-TolC, EmrAB-TolC, EmrKY-TolC, and MacAB-TolC. These pumps belong to three classes of transporters: RND, MFS and ABC, which again indicates the need for the simultaneous presence of both ATP-dependent and voltage-dependent pumps. A TolC-containing transporter, such as AcrAB-TolC, consists of the outer membrane channel TolC (common to all pumps), the multidrug efflux pump membrane fusion lipoprotein AcrA (common to AcrAB-TolC and AcrAD-TolC pumps), and the inner membrane multidrug efflux pump RND permease AcrB. The AcrA adapter protein binds the AcrB and TolC proteins to each other and prevents substrates from entering the intermembrane space. Since the TolC protein is the same for all pumps, the possibility of temporary connection through adapter proteins of TolC alternately with one or the other pump, depending on cellular needs, is not ruled out. It is during these possible pump changes that the TolC channel opens into the periplasmic space. Thus, hypothetically it could be possible to assume the existence of four ways of penetration of substances into the periplasmic space.

**Figure 4:**
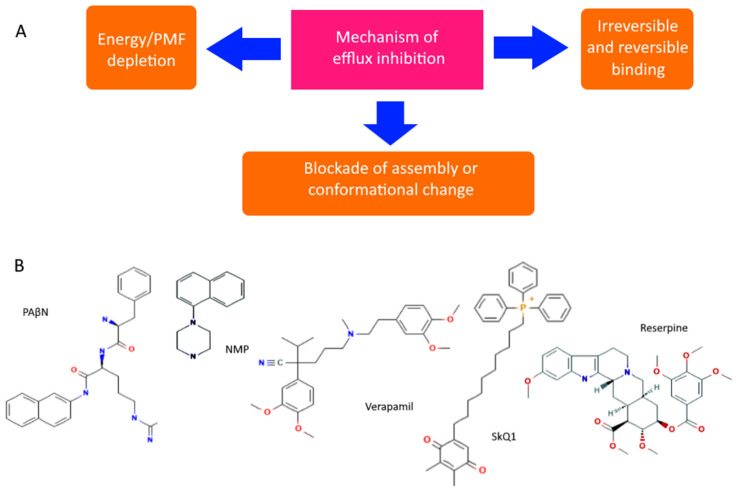
Diagram showing the main mechanisms of overcoming non-specific protection due to inhibition of MDR pumps (**A**) and some MDR pump inhibitors (**B**).

However, even non-specific protection can be disabled or its impact reduced. There are both specific and natural compounds that reduce the efficiency of pumps [92,93]. Although there are quite a few hypothetical mechanisms for reducing pump performance, the most realistic seem to be: (1) competitive or irreversible binding to the active site, preventing the formation of active transporters on the membrane, or blocking conformational changes (open/closed state), and (2) a decrease in membrane potential and ATP level. All known pump inhibitors interfere with MDR pumps by one of these mechanisms, some of which are presented in Figure 4. PAβN (Phenylalanine-Arginine-β-napthylamide) is a competitive inhibitor and affects membrane permeability [94,95]. NMP (1-(1-napthylmethyl)-piperazine) and SkQ1 (10-(plastoquinonyl)decyltriphenylphosphonium) are inhibitors of AcrAB-TolC efflux pumps. The mechanism of action of NMP remains unknown, while the mechanism of action of SkQ1 is well known. SkQ1 is a competitive inhibitor, and also reduces the membrane potential, thus affecting the bioenergetics of bacteria [14,15]. Verapamil is a well-known channel blocker that directly binds with the efflux pumps and inhibits the efflux of antibiotics [96]. If the substances discussed above are synthetic, then reserpine refers to indole alkaloids of plant origin. The main targets of reserpine are the MFS and RND pumps, with which it directly binds and inhibits their work [93].

It should be noted that although under the action of antibiotics there may be upregulation of pump expression, increasing the number of individual pumps responsible for their efflux, but it is unlikely that bacteria have mechanisms to reduce nonspecific protection. Therefore, the mechanism of suppression of the genes encoding the pumps is rather hypothetical, and the decrease in the expression level can be explained either by a general decrease in bacterial bioenergetics and biosynthesis, or by nonspecific binding to mRNA.

## 5. Role of MDR Pumps in Biofilm Protection and Bacterial Persistence

A bacterial population usually consists of two subpopulations, (1) planktonic and (2) sessile, between which there is an equilibrium maintained by various factors, such as quorum sensing signals [97] or electrical signals [98]. The resistance of bacteria in the biofilms (in form of MIC, MBC) can exceed the resistance of planktonic forms by two orders of magnitude [99]. The formation of biofilms includes several stages: (1) reversible attachment of planktonic cells to the surface, (2) cell proliferation and formation of microcolonies, (3) transformation of colonies into clusters of multilayer cell formations synthesizing extracellular polymeric substances (EPS), which form a matrix, (4) maturation of the biofilm with the transition of some cells to the planktonic form for the formation of new colonies [100]. Biofilms can be formed by different types of bacteria, which has its own advantages, both metabolically and in terms of protection. The colonial state of bacteria in the form of biofilms provides many advantages including (1) a variety of habitable conditions, (2) improved resource absorption, (3) cooperation between participants, (4) increased protection and regeneration, (5) accelerated genetic exchange, (6) external enzymatic systems, and therefore is in many ways more beneficial for bacteria [101]. Unfortunately for them, there is a serious drawback in the existence of such a “pseudo-multicellular” form: due to crowding, in comparison with planktonic forms, ingress of bacteriophages leads to a very rapid elimination of the entire colony, therefore, to protect against bacteriophages, bacteria use defense systems such as the CRISPR system and retrons [102,103,104].

At the same time, the role of MDR pumps in the formation of biofilms is quite significant: (1) transportation of EPS outside the cell for matrix formation, (2) export of quorum sensing and quorum quenching signals, (3) promoting or preventing adhesion to surfaces and other cells, (4) protection from toxins, antibiotics, and toxic metabolites resulting from coexistence in a limited volume of biofilms.

Other mechanisms of protection against antibiotics include absorption of antibiotics by bacterial cells at the biofilm boundary, the death of which is both protective [77,78] and a source of nutritional resources for the population inside the biofilm, which uses them for the so-called necrotrophic growth [105]. So, it can be summarized that antibiotics may not be effective enough for sessile bacterial culture in biofilm, and some antibiotics, such as streptomycin and chloramphenicol, even cause increased biofilm formation [106,107]. At the same time, MDR pumps not only play an important role, pumping antibiotics out of cells, but also creating a certain microenvironment that affects the entire population around [108].

Another aspect of the protection of bacteria from antibiotics is the transition to a special state of persistence, when bacteria become tolerant to antibiotics, regardless of their concentration [109,110,111]. The persistence of bacteria is caused by stress and antibiotics [112], which determines the effect of MDR pumps on the formation of persister cells [113]. Taking into account that one of the mechanisms of persistence is membrane depolarization under the action of the TisB toxin [114,115,116], it is in this case that a state arises when the cell is deprived of the potential on the membrane, and the energy provided by membrane-independent energy processes (for example, glycolysis) can power ATP-dependent pumps to expel harmful substances when the rest of the pumps are disabled due to a lack of potential on the membrane.

## 6. Bacteriophage as a Natural Antibacterial Weapon

Bacterial viruses (bacteriophages or phages) are the largest known group of viruses containing predominantly double-stranded genomic DNA, although there are small groups of phages with single-stranded DNA, as well as double- and single-stranded RNA [31].

There are approximately 10^32^–10^33^ phages in the world [117], and they play an important role in the regulation of the global bacterial population. For instance, bacteriophages are responsible for the death of 20–40% of marine bacteria daily [118]. Without a doubt, bacteriophages are more specific to particular strains of bacteria, which has its advantages. This fact has found its application in phage therapy, which causes much less damage to the body than antibiotic therapy [119,120]. However, under conditions where the infection is caused by a number of different bacteria, as occurs with wound infections, bacteriophages are significantly less effective than antibiotics [121].

Unlike the bacterium-antibiotic system, the bacterium-bacteriophage system is an evolving one. Natural selection prevails over the population of bacteria and bacteriophages. In the bacterium-antibiotic system, only the bacterium evolves, in contrast to the bacterium-bacteriophage systems, or the bacterium and the antibiotic producer. On the one hand, there is geographic separation of bacteriophages [122], and bacteriophages from one geographic region may not affect bacteria from another region. On the other hand, a wide geographical distribution of phages capable of lysing the same isolate is well known [123]. For example, bacteriophages obtained in Russia may not have strong effect on patients in Bangladesh with diarrhea caused by *E. coli* [124], and cross-infection of *E. coli* bacteria with bacteriophages obtained in Mexico and Bangladesh showed a high specificity of phages to bacteria from their own region [125]. This seems to be especially surprising, given the fact that, for example, plant viruses can replicate in mammalian cells [126], which indicates the important role of a virus recognizing its potential target. This can be explained by the presence in the phage genome of a large proportion of variable genes with a potential role in host recognition, which suggests that these regions are involved in phage adaptation to diverse bacterial hosts [127].

Thus, a viral infection begins with the recognition of a receptor on the cell surface by the virus. Bacteriophages belong to several families: Corticoviridae, Cystoviridae, Inoviridae, Fuseloveridae, Lipothrixviridae, Leviviridae, Microviridae, Myoviridae, Plasmaviridae, Podoviridae, Rudiviridae, Siphoviridae, and Tectiviridae, differing also in receptor groups, which are subdivided into (1) proteinaceous receptors; (2) sugar moieties of polysaccharides; and (3) combination of proteins and sugar moieties [128,129,130,131]. Adsorption depends on several physicochemical parameters, such as pH, ionic composition of the medium, temperature, and proceeds in two stages: first, reversible binding occurs, and then binding becomes irreversible [132]. Penetration of the nucleic acid occurs after irreversible binding. Penetration occurs due to lysis of the peptidoglycan layer under the action of phage lysozymes (“lysis from outside”) and is individual for each phage [133]. Factors such as membrane potential and ATP level may be necessary for a successful bacteriophage infection [134,135]. In this case, apparently, it is the potential, and not the pH gradient, that is important for the formation of the bacterium–bacteriophage complex [136]. However, this issue is still debatable.

## 7. Mechanisms of Phage Penetration into a Bacterial Cell

Despite the rather long time of astonishment of bacteriophages, so far the mechanism of their penetration has been less studied than the mechanisms of penetration of eukaryotic viruses. In general, prokaryotic viruses are tailed viruses in which the capsid is attached to the tail. The viral genome in the capsid is packed so tightly that a pressure of up to 60 atmospheres is created for some bacteriophages, which may be the main driving force for injecting their genetic materials into the cytoplasm of the host cell [137]. At the same time, it is rather doubtful that all energy accumulated during packaging is stored in the nucleic acid or capsid. There are several models of bacteriophage nucleic acid injection: (1) the continuum mechanics model [138]; and (2) hydrodynamic model of in vitro DNA ejection [139]. Bacteriophages can eject DNA either momentarily, as in the case of the lambda phage, or discontinuously, as in the case of the T5 phage. Moreover, in some cases, host cell proteins are also involved in this process, thus phage T7 DNA transport into the cell is thus governed by enzymes and is independent of any physical force or pressure [137]. For dsRNA phages, which contain phospholipids and proteins, entry occurs by membrane fusion, allowing the capsid to enter the cell [140].

At the same time, the mechanism of penetration of tailed phages, especially T4 phage, remains the most studied [141]. The process begins with receptor recognition and irreversible binding to it. Baseplate undergoes a large conformational change from a high-energy dome-shaped structure to a low-energy star-shaped structure, short tail fibers rotate downward about 90° and this defines irreversible binding. Then there is a series of successive rearrangements of the basal plate, sheath and tail tube, provides the required motion for the needlelike tip of the tail tube to penetrate the cell membrane. The hardest part is to penetrate the peptidoglycan layer. The bacteriophage needs to penetrate the peptidoglycan layer twice: (1) during the initiation of infection; and (2) during the release of virions from the cells. At the stage of virion release, the bacteriophage uses the expression products of late genes, such as the genes for endolysins and holines, small hydrophobic hole-forming proteins [10,31,142]. But at the stage of infection initiation, to penetrate the cell, the phage is forced to use capsid-associated phagolysin or to use some other mechanism for penetrating the peptidoglycan layer. Therefore, to get inside the cell, the phage has to locally disrupt the peptidoglycan layer by means a capsid -associated phage lysin, such as the gp5 baseplate protein produced by the T4 bacteriophage [31,143]. An alternative to this may be the use of outer membrane porins that permeate the peptidoglycan layer. At the same time, the presence of capsid-associated lytic enzymes is not known for all phages, which suggests the presence of another penetration pathway, possibly due to proteins penetrating the peptidoglycan layer.

## 8. MDR Pumps as Phage Receptors

If we consider bacteriophage receptors, then we can notice a certain regularity, namely, that the majority of receptors in gram-positive bacteria, as expected, refer to the components of the cell wall and the peptidoglycan layer, with the exception of the GamR protein [128], the function of which is not completely clear, and which appears to be related to cobalt transporter proteins belonging to the ATP-binding cassette (ABC) family [144]. Gram-negative bacteria have significantly more peptide receptors, which are either related to porins, such as OmpC and OmpF, or channels, such as LamB and FadL, or pumps and transport systems, such as BtuB, FhuA, TonB, and TolC [128,145]. It could be assumed that all proteins that are localized on the outer membrane and the LPS layer could be receptors, but these receptors make up 79% (108 out of 137) of the proteins exposed on the outer membrane [146]. Moreover, none of the included genes belong to the essential genes, while among the non-included 33% (7 out of 29) belong to the essential genes, which indicates their possible connection not only with the location on the outer membrane, but with certain functional features.

The adsorption of bacteriophages to the surface of bacteria is usually described by several stages: (1) initial contact, (2) reversible binding, and (3) irreversible attachment [128,147]. Therefore, the question arises about the relationship between receptors (porins, pumps, channels) and the ability of bacteriophage nucleic acids to penetrate into cells [148]. Whether bacteriophages use the structural features of receptors for penetration through the outer membrane or only for recognition and attachment to the surface of bacteria remains debatable. In this regard, of particular interest are pumps localized on the outer and inner membranes, such as pumps from the TolC-containing pump group and the CusCFBA copper/silver efflux system.

A bacterial cell envelope is a barrier in the way of bacteriophage.

At the same time, phage binding to the receptor, which is a porin, can cause changes in the transport properties of the channel. Thus, when the lambda phage was bound to maltoporin, even though the kinetics of sugar binding did not change, ion fluxes through the pores of maltoporin in the phage–receptor complex share a new common pathway [149]. These data directly indicate that the irreversible binding of the phage to the receptor with the formation of the MDR pump-phage complex apparently leads to a change in the way the MDR pumps for expel xenobiotics.

In this regard, another question arises about phage-antibiotic synergy (PAS) [150] in the context of the use of MDR pumps by both parties. Obviously, this largely depends on the properties of the antibiotic and bacteriophage used [151,152]. If an antibiotic lowers the potential on the membrane that the phage needs to penetrate, then their combined use seems impossible, but in this case, sequential use can provide the necessary synergy. Therefore, the main aspects of successful PAS are (1) the choice of the combination of antibiotics; and (2) the correct sequence of actions in combination therapy. It is not entirely clear whether bacteriophage binding to pumps affects their work on pumping out their substrates, and whether pumping out the substrates at the moment of binding affects the binding process itself. Without answers to these questions, the future prospects of PAS look uncertain, varying from phenomenal success to only limited use [152,153,154,155,156]. It is necessary to pay attention to another important aspect of the interaction between phages and antibiotics. That in the case of antibiotic therapy and in the case of phage therapy, disruptive selection takes place, in which conditions favor several extreme variants of variability, but do not favor an intermediate, average state. However, these extreme states are not the same for phages and antibiotics, so a synergistic effect is very likely.

## 9. Conclusions

It is no exaggeration to say that MDR pumps are the cornerstone and a prerequisite for almost any bacterial cellular process. Pumps play a key role in xenobiotic defense and attack processes by participating in the export of toxins; they are a key factor in the formation of biofilms, and make an important contribution to bacterial persistence. Even in the absence of specialized antibiotic defense systems, the very presence of these highly regulated systems provides a high level of protection, allowing bacteria to survive at antibiotic concentrations several orders of magnitude higher than what would kill them if they did not have such a defense system. This allows us to state that the system of bacterial MDR pumps is a nonspecific defense against xenobiotics, which determines the basic, primary resistance, “immunity” of bacteria to toxins, antibiotics and other substances that negatively affect bacterial cells. Other resistance mechanisms (drug target modification, drug inactivation, target switching, and target sequestration) are secondary, specific mechanisms that, when combined with the primary system, determine the existence of a phenomenon called super-resistant bacteria. A long-term strategy to combat such bacteria cannot be made possible by simply bypassing specific defenses without paying due attention to overcoming non-specific defenses.

The bacterial MDR pump system appears to have evolved from the last universal common ancestor (LUCA), in conjunction with bacteriophages. Just as bacteriophages use the host’s metabolic and biosynthetic systems, they use the bacteria’s need for MDR pumps which they utilize to bind to bacterial cells and then enter them. Although MDR pump systems are well optimized for pumping out xenobiotics and are rather conservative [157], they undergo many mutations under the action of bacteriophages [158], which leads to a decrease in the efficiency of antibiotic pumping and a decrease in the contribution of nonspecific protection to overall resistance. The study and understanding of these processes will allow us to either seriously delay the onset of the post-antibiotic era or even prevent it altogether in the future.

## Figures and Tables

**Table 1:** The role of MDR pumps in antibiotic resistance. Antibiotics groups by mechanism of action.

Mechanism of Action	Antibiotic Group	Antibiotics ^1^	References ^2^
Protein synthesis: 30S ribosomal subunit binding	Aminoglycosides	Kan, Sm, Gm	[54,55]
	Tetracyclines	Tet, Dox, Min	[53,56]
Protein synthesis: 50S ribosomal subunit binding	Streptogramins	Q/D	[57]
	Macrolides	Erm, Clr, Azm	[53,58,59]
	Oxazolidinones	Tzd, Rzd	[60]
	Lincosamides	Lnm, Cdm, Prm	[61,62,63]
	Chloramphenicol		[53]
Nucleic Acid Synthesis	Quinolones and Fluoroquinolones	Cip, Lvx, Nal	[56]
Metabolic Pathways	Sulfonamides	Sul, Smx	[53,64]
	Trimethoprim		[64]
Depolarize Cell Membrane	Lipopeptides	Dap	[65]
	Lantibiotics	Gdm	[65]
Cell Wall Synthesis	β-Lactams	Amp	[53]
	Carbapenems	Imp	[66]
	Cephalosporins	Cpx	[67]
	Monobactams	Azn	[68]
	Glycopeptides	Van	[69]

^1^ Abbreviations: Amp, ampicillin; Azm, azithromycin; Azn, aztreonam; Cdm, clindamycin; Cip, ciprofloxacin; Clr, clarithromycin; Cpx, cephalexin; Dap, daptomycin; Dox, doxycycline; Erm, erythromycin; Gdm, gallidermin; Gm, gentamicin; Imp, imipenem; Kan, kanamycin; Lnm, lincomycin; Lvx, levofloxacin; Min, minocycline; Nal, nalidixic acid; Prm, pirlimycin; Q/D, quinupristin/dalfopristin; Rzd, radezolid; Sm, streptomycin; Smx, sulfamethoxazole; Sul, sulfacetamide; Tet, tetracycline; Tzd, tedizolid; Van, vancomycin. ^2^ MDR pumps play an important role in the non-specific protection cells from antibiotics, and for many of them (probably for all) there are corresponding pumps that remove them from cells, which is confirmed by the reference to the removal by MDR pumps of the most famous representatives of antibiotic groups.
